# Effects of High-Fat and High-Fat High-Sugar Diets in the Anxiety, Learning and Memory, and in the Hippocampus Neurogenesis and Neuroinflammation of Aged Rats

**DOI:** 10.3390/nu15061370

**Published:** 2023-03-11

**Authors:** Bárbara Mota, Miguel Ramos, Sandra I. Marques, Ana Silva, Pedro A. Pereira, M. Dulce Madeira, Nuno Mateus, Armando Cardoso

**Affiliations:** 1 Unit of Anatomy, Department of Biomedicine, Faculty of Medicine, University of Porto, Alameda Prof. Hernâni Monteiro, 4200-319 Porto, Portugal; 2 NeuroGen Research Group, Center for Health Technology and Services Research (CINTESIS), Rua Dr. Plácido da Costa, 4200-450 Porto, Portugal; 3 Department of Chemistry and Biochemistry, Faculty of Sciences of University of Porto, Rua do Campo Alegre, 4169-007 Porto, Portugal; 4 UCIBIO—Applied Molecular Biosciences Unit, Laboratory of Toxicology, Faculty of Pharmacy, University of Porto, 4050-313 Porto, Portugal; 5 2i4HB—Institute for Health and Bioeconomy, Faculty of Pharmacy, University of Porto, 4050-313 Porto, Portugal; 6 CINTESIS@RISE, Faculty of Medicine, University of Porto, Alameda Prof. Hernâni Monteiro, 4200-319 Porto, Portugal

**Keywords:** elderly, brain aging, high-caloric diets, hippocampus, learning and memory, astrocytes, neurogenesis, neuroinflammation, neurodegeneration

## Abstract

High-caloric diets induce several deleterious alterations in the human body, including the brain. However, information on the effects of these diets on the elderly brain is scarce. Therefore, we studied the effects of 2 months of treatment with high-fat (HF) and high-fat-high-sugar (HFHS) diets on aged male Wistar rats at 18 months. Anxiety levels were analyzed using the open-field and plus-maze tests, while learning and memory processes were analyzed using the Morris water maze test. We also analyzed neurogenesis using doublecortin (DCX) and neuroinflammation using glial fibrillary acidic protein (GFAP). In aged rats, the HFHS diet impaired spatial learning, memory, and working memory and increased anxiety levels, associated with a reduction in the number of DCX cells and an increase in GFAP cells in the hippocampus. In contrast, the effects of the HF diet were lighter, impairing spatial memory and working memory, and associated with a reduction in DCX cells in the hippocampus. Thus, our results suggest that aged rats are highly susceptible to high-caloric diets, even if they only started in the elderly, with an impact on cognition and emotions. Furthermore, diets rich in saturated fats and sugar are more detrimental to aged rats than high-fat diets are.

## 1. Introduction

Diet choice is one of the most important factors for improving health, with special relevance to brain health and cognitive function [1,2,3]. One of the problems of modern society is that the consumption of high-caloric diets rich in fat, sugars, and salt has increased in recent years in developing and developed countries. It is commonly known as the Western Diet (WD). Together with the imbalance between consumed and expended calories, it is known to lead to overweight or obesity, which had reached pandemic proportions by the 21st century [4,5,6,7]. Given this, it is important to know the spectrum of alterations potential induced by high-caloric diets. 

Another issue of contemporary societies is the increasing aging population. Aging, together with obesity, are responsible for more than 80% of deaths in developed countries [5]. It is well known that aging is often associated with cognitive dysfunction, typically attributed to natural brain atrophy [8,9]. However, this process can be influenced and accelerated by health-related biological and nonbiological factors, such as nutritional imbalances. Nutrition has recently been identified as a modulating factor, particularly in middle-aged individuals [8,10]. Recent studies have established that chronic consumption of WD during midlife is associated with a higher prevalence of dementia development, with impairments in learning and memory processes, both in humans and rodents [2,9,11,12,13,14,15]. These findings have heightened scientific interest in the effects of nutrition on brain function, with a focus on the elderly [1]. The impaired learning and memory capacity observed in humans and rodents after WD consumption, even for short periods, resulted, at least in part, in hippocampal alterations [5,16]. Thus, it is important to understand the effects of high-caloric diets on the brain during aging.

It is well known that the hippocampus plays a crucial role in learning and memory processes and is one of the brain regions most vulnerable to metabolic changes induced by WD consumption, including impaired neurogenesis, synaptic function, neuronal growth, and dendritic integrity [1,8,9,12,17]. Animal studies have shown that consuming high-caloric diets may affect hippocampal-dependent learning and memory, a risk factor for dementia and milder cognitive impairment [5,11,12,13,16,17,18]. Beyond neurogenesis, WD consumption is also associated with neuroinflammatory processes [5]. 

Neuroinflammation is characterized by the activation and proliferation of glial cells after harmful stimuli or injuries to the brain and is known to be involved in age-related cognitive impairment [2,15]. Astrocytes play a major role in neuroprotection and brain homeostasis and respond to all forms of insults through reactive astrogliosis, characterized by pronounced hypertrophy, and astrocytosis, an increase in the number of proteins and mRNA of the glial fibrillary acidic protein (GFAP) marker [8,9]. Recent studies have shown that short periods of high-caloric diet consumption induce astrogliosis, suggesting that these animals are sensitive to dietary factors [11]. Inflammation in the brain has recently been linked to cognitive dysfunction and neuroinflammatory markers in the hippocampus, which precede hippocampal dysfunction [7,11,18]. It seems that the natural process of aging and the consumption of WD induce a shift in the brain where the naturally occurring pro-inflammatory state is exacerbated, leading to neurodegeneration and cognitive decline [1,2]. 

Neurogenesis is the ability to produce new neurons throughout life. It is well known that aging suppresses neurogenesis, leading to impairments in learning and memory [19,20]. Behavioral studies have shown a positive correlation between the degree of neurogenesis and the performance of hippocampal-dependent memory tasks in rodents [21]. Neurogenesis is also involved in processes related to mood regulation, such as anxiety and depression [21,22]. Adult hippocampal neurogenesis is a key element of functional hippocampal integrity during aging [21]. Beyond aging, consuming high-caloric diets also decreases neurogenesis because consuming fat can reduce cellular proliferation in the hippocampus [9]. 

Despite the known detrimental effects of high-caloric diets on the hippocampus, few studies have analyzed the effects of high-caloric diets in elderly individuals. Indeed, given that the percentage of older people is gradually increasing in contemporary society, it is crucial to understand better the spectrum of potential alterations induced by high-caloric diets in the brains of older individuals. Indeed, there is a lack of studies aiming to understand the effects of high-caloric diets in elderly rats and documenting hippocampal morphological alterations related to neuroinflammation. Ultimately, this study will help to create evidence that future human diets may be optimized to avoid deleterious effects such as neuroinflammatory processes. Furthermore, because the source of calories in Western diets is mainly fat and sugar, it is important to know the impact of fat and the combination of fat and sugar. Thus, trying to bridge these issues, this study investigated two different high-caloric diets frequently consumed by humans and analyzed their effects on male rats aged 18 months. In particular, we compared the effects of a saturated fat-rich diet with those rich in refined sugar and saturated fat. After diet treatments for 8 weeks, rats were subjected to a sequence of behavioral tests for 4 weeks to explore their impacts on spatial learning and memory, anxiety, and locomotor activity. Furthermore, we evaluated whether there was a relationship between the possible behavioral changes induced by these two high-caloric diets and hippocampal neurogenesis and neuroinflammation.

## 2. Materials and Methods

### 2.1. Animals

A total of 30 (thirty) adult male Wistar rats (*Rattus norvegicus*), bred at the Faculty of Medicine of the University of Porto, Portugal animal facility and aged approximately 18 months, were maintained with water and food *ad libitum.* The temperature, humidity, and lightning (12 h dark/light cycle) were controlled. All animals were housed 2 per cage to allow weekly liquid and food consumption quantification and to avoid social isolation. To minimize stress caused by handling, the bedding was changed on the same days that animals were weighed, which occurs once per week. The experiments were made following the guidelines of the European Communities Council Directives of 22 September 2010 (2010/63/EU) and Portuguese Act n°113/13, and approved by ORBEA, the internal committee of the Faculty of Medicine of the University of Porto, Portugal. During the whole experimental period, the health of animals was monitored to reduce the number of animals used as well as stress and suffering. 

### 2.2. Diets

The rats were divided into three groups with different diets, as shown in Table 1. Taking into account the advanced age of the animals, a few weeks after starting the diet treatment, two animals died, and four animals reached the human endpoints and were unable to proceed with the study. The control diet (n = 8)—free access to tap water and standard laboratory chow (Mucedola 4RF21; proteins (18.5%), fat (3%), and fibers (6% fiber). High-fat diet (HF) (n = 10)—special Western laboratory chow (ssniff^®^ E15749—34; proteins (21.2%), fat (20.1%), sugar (14%), fibers (5.5%), and free access to tap water. High-Fat High-Sugar diet (HFHS) (n = 6)—special Western laboratory chow (ssniff^®^ E15749—34; proteins (21.2%), fat (20.1%), sugar (14%), and fibers (5.5%), and unrestricted access to a 15% sucrose aqueous solution. Diet and liquids were accessible *ad libitum* throughout the experiment period (12 weeks) and replaced weekly. Food and fluid intake was determined every week.

### 2.3. Behavioral Assessment

Before beginning behavioral tests, the rats were handled for five consecutive days. Behavioral tests were performed after at least 30 min of familiarization with animals in the space. Testing was performed at the same time, beginning at 13:00 h. During the behavioral test period, animals were maintained on a group-specific diet.

#### 2.3.1. Morris Water Maze (MWM) Test—Reference Memory

The maze was made of a black circular pool (180 cm diameter, 50 cm depth) positioned in the center of a room having extra maze cues [23]. The pool was filled with water (21 ± 1 °C) and virtually and equally divided into four quadrants. A black escape platform, 10 cm in diameter, was positioned at the center of one of the quadrants and 2 cm below the water surface. The swim path was recorded using a computerized video tracking system (EthoVision XT 8.5; Noldus, The Netherlands). In the place-learning task, animals were trained to find and climb onto a submerged escape platform. The rats were subjected to two daily trials for 14 consecutive days for acquisition. Before trials, a pseudorandom order was used to achieve the starting position but ensured that each position was used once in each block of four trials.

The animals were placed in the water, always facing the pool wall. After the 60 s trial, the rats that did not achieve the platform were guided by the experimenter and allowed to remain there for 15 s—finished the first and third trials of each block, a clean cage was used to place the animals and will enable them to rest for a 30 s interval before the beginning of trials 2 and 4. The location of the platform did not change during the acquisition period. The swim path length was measured for each trial. On the next day, the conclusion of the acquisition, the animals were subjected to a single 60-probe trial without the platform. The number of passages in the zone where the platform was located (platform crossings) was considered a measure of the accuracy in recalling the previous platform position. The time spent swimming in the target quadrant was also analyzed. One day after the conclusion of the probe trial, all rats were tested on the visible platform task to evaluate their sensorimotor ability. In this task, the rats were given one block of four trials separated by 30 s inter-trial intervals. The platform, which was painted white, was exposed at 3 cm above the water surface. Before every trial, the platform was moved to a different position. The distance swum used to locate the platform was recorded and averaged across four trials.

#### 2.3.2. Morris Water Maze (MWM) Test—Working Memory

One day after the sensorimotor ability task, the animals were tested on a delayed match-to-place version [24] of the Morris water maze to analyze spatial working memory. Through the next three days, they received two trials per day. In the first trial (information trial), the platform was in a novel position different from the quadrant and distant from the edge of the maze from its placement in the reference memory task. Each rat was placed in a pool facing the wall, distal to the platform. If the rat did not find the platform after 60 s, the experimenter guided the rat to the platform, remaining there for 15 s. In the second trial (retention trial), the submerged platform remained in the same position as in the information trial. The starting position was constantly distal from that of the platform. Otherwise, the trials were performed in the same manner as the spatial reference memory task. A 1 min inter-trial interval was permitted between the information and retention trials.

#### 2.3.3. Open-Field Test

The open-field [25,26] apparatus comprised a white acrylic arena measuring 100 × 100 × 40 cm. Animals were introduced at the corners of the apparatus and tested for 5 min. The time that animals spent in the outer zone of the open field, defined as 20 cm from any wall, and in its inner zone, defined as a 60 × 60 cm square in the center of the arena, was calculated using a computerized video tracking system (EthoVision XT 8.5, Noldus, The Netherlands). The total distance traveled by the animals during each session was also calculated. The apparatus floor was constantly cleaned and dried between animals. 

#### 2.3.4. Elevated Plus-Maze Test

The elevated plus-maze [26] apparatus consisted of a black acrylic cross with two opposite open arms, and two opposite closed arms (50 × 12 cm) joined with a common central square (12 × 12 cm). High walls of 50 cm in height enclosed the close arms of the apparatus. In every trial, animals facing one of the closed arms were placed on the central square. The duration of the acquisition was 5 min, during which animals were allowed to explore the apparatus. The behavior of the rats was recorded and analyzed using a computerized video tracking system (EthoVision XT 8.5, Noldus, The Netherlands). The time spent by the rats in the open arms closed arms, and the central square was calculated. The total distance traveled by the animals during each session was also measured. At the end of each session, the apparatus was thoroughly cleaned and dried.

### 2.4. Perfusion and Tissue Processing

After the behavioral assessment, half of the animals from each group were perfused through the aorta with calcium-free Tyrode’s solution (0.12 M NaCl, 5.4 mM KCl, 1.6 mM MgCl_2_▪H_2_O, 0.4 mM MgSO_4_▪H_2_O, 1.2 mM NaH_2_PO_4_▪H_2_O, 5.5 mM glucose and 26.2 mM NaHCO_3_), followed by 4% paraformaldehyde (PFA) in 0.1 M phosphate buffer saline (PBS). Before perfusion, samples of blood were collected through cardiac puncture. Brains were collected and post-fixed for 2 h in 4% PFA, followed by immersion in 10% sucrose in 0.1 M PBS. The three types of fat (abdominal, adrenal, and gonadal) were collected, post-fixed overnight in 4% PFA, and then immersed in 70% alcohol. The blocks of the brain were mounted on a vibratome, serially sectioned in the coronal plane at a thickness of 40 µm and collected in PBS. A set of vibratome sections containing the hippocampal formation was selected from each brain using a systematic random sampling procedure for immunostaining in a proportion of 1:12 for each set, GFAP and Doublecortin (DCX).

### 2.5. Immunohistochemistry

For immunohistochemistry, selected brain sections were washed three times in PBS, treated with 3% H_2_O_2_ solution for 7 min to inactivate endogenous peroxidase, washed three times in PBS containing 0.5% Triton (T), and incubated for 72 h at 4 °C for GFAP (Z0334, Dako, 1:4000 in PBS 0.5%T) and DCX (E-6, sc—271390, Santa Cruz Biotechnology, 1:400 dilution in PBS 0.5%T). The sections were then washed thrice in PBS 0.5%T and incubated with the respective biotinylated secondary antibodies (Goat anti-rabbit BA—1000, 1:400 dilution in PBS 0.5%T, for GFAP; and Horse anti-mouse BA—2001, 1:400 dilution in PBS 0.5%T, for DCX, both from Vector Laboratories). The sections were treated with an avidin-biotin-peroxidase complex (Vecta-stain Elite ABC kit, Vector Laboratories; 1:800 dilution in PBS with 0.5%T). The last two incubations were performed at room temperature for 1 h. After treatment with the peroxidase complex, sections were incubated for 10 min in 0.05% diaminobenzidine (Sigma-Aldrich, Missouri, USA) to which 30 µL of 0.01% H_2_O_2_ solution was added. The sections were rinsed with PBS for at least 15 min between each step. 0.5%T X-100 was added to PBS for all immunoreactions and washing steps to increase tissue penetration. The specificity of the immune reactions was controlled by omitting the incubation step with primary antiserum. All immunohistochemical reactions and washings were performed in 12-well tissue culture plates to ensure that the staining of sections from all groups was performed in parallel and under identical conditions. The sections were mounted on gelatin-coated slides, air-dried, dehydrated, and cover-slipped using Histomount (National Diagnostics, Atlanta, USA).

### 2.6. Quantification of Astrocyte Morphology

Immunostained brain sections were photographed using a light microscope (Zeiss Scope A.1) equipped with an AxioCam MRc5 camera at a final magnification of 20×. The layer boundaries of the DG, CA3, and CA1 were consistently defined at all levels along the septotemporal axis of the HF based on cytoarchitectonic criteria [27] and using a rat brain atlas [28]. In addition, astrocytes belonging to the CA2 hippocampal field were included in the CA3 region. The pictures obtained from the AxioVision Rel 4.8 program were used in the ImageJ program and run on a macro [29] modified to count astrocytes [30] to assess the number of GFAP-IR (immunoreactive) astrocytes as well as the length of their processes and soma. 

### 2.7. Quantification of Areal Density of DCX-Immunoreactive Cells

Immunostained brain sections were analyzed and drawn using a light microscope equipped with a *camera lucida* at a final magnification of 160×. Estimates for DCX cells were obtained from the subgranular layer of the dentate gyrus. Cells were considered immunoreactive for DCX when they displayed darkly stained *perikarya*. The subgranular layer was consistently delineated at all levels along the septotemporal axis of the hippocampal formation based on cytoarchitectonic criteria [31], and by using a rat brain atlas [28], the subgranular layer was further defined as an approximately 30 μm thick ribbon of tissue between the granular layer and the hilus [32]. The estimates for each cell type were obtained from an average of 12 immunostained sections per rat sampled, as described above. The calculation of layers areas was made using drawings obtained from *camera lucida*. A transparent sheet bearing a test system composed of a set of regularly spaced points was overlaid on the drawings, and the number of points that fell within the limits of the subgranular layer was counted. The area of each layer was estimated by multiplying the number of points that fell within limits by the value of the area per point of the test system (0.0096 mm^2^). The cell numbers obtained were divided by the values of the corresponding laminar areas to yield areal density values (number of cells/mm^2^).

### 2.8. Data Analysis

Results from the behavioral test are expressed as mean ± SEM and morphological data as mean ± SD. GraphPad Prism software (version 8.0.2) was used to perform graphics and the respective statistical analyses. Repeated measures analysis of variance (ANOVA) was applied to analyze data from the distances traveled in the Morris water maze and distances traveled in spatial working memory. Two-way ANOVA was applied to analyze the effect of treatment and zone in the open-field and elevated plus-maze tests and to analyze the impact of treatment on time spent in the opposite quadrants and the target one of the Morris water mazes. Data from the other behavioral tests, body weight, total number of DCX-IR neurons, and number and morphology of astrocytes were analyzed with one-way ANOVA, applying treatment as the independent variable. Whenever appropriate, ANOVA was followed by Tukey’s (HSD) *post-hoc* comparisons. In all statistical analyses was considered *p* < 0.05 as the level of significance.

## 3. Results

### 3.1. Body Weight

**Figure 1:**
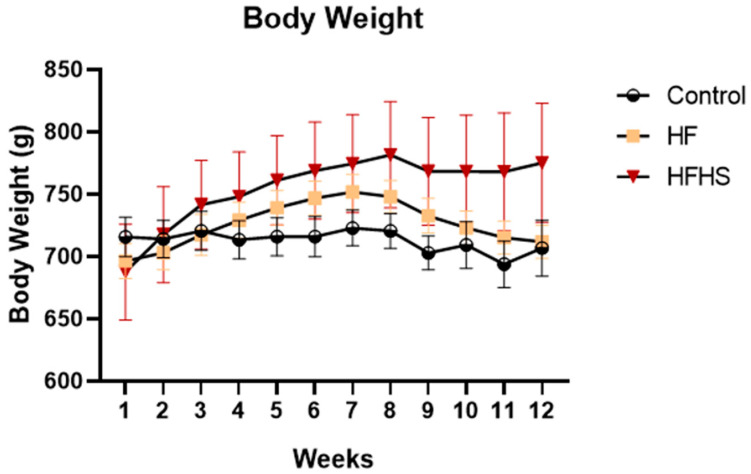
The graphic shows the body weight increase, in grams per week (mean ± SEM), in control (control), high-fat (HF), and High-Fat High-Sugar (HFHS) groups across the entire experiment.

The body weight of the animals, shown in Figure 1, was recorded every week during all the experiments to control and assess whether the animals became overweight or lost weight, as these two points are considered relevant for our study and the human endpoints, according to the European Communities Council Directive 2010/63/EU. The overall caloric consumption (Table 2), on average, per group during the 12 weeks of treatment, expressed in Kcal (SEM), was 14,032 (546.2) for control animals, 16,335 (411.7) for HF animals, and 23,311 (3453) for HFHS animals. There was a significant effect of dietary treatment on Kcal consumption (F (2, 9) = 8.927, *p* < 0.01). HFHS rats consumed more calories than controls (*p* < 0.01) and HF rats (*p* < 0.05). Moreover, HF consumed more calories than controls (*p* < 0.05). Before the diet intervention, body weights, expressed in g (SEM), were 698.6 (17.23) for control animals, 641.3 (14.11) for HF animals, and 664.8 (32.17) for HFHS animals. After dietary treatment, body weights expressed in g (SEM) were 723.4 (27.31) for controls, 715.6 (13.49) for HF, and 725.2 (85.8) for HFHS animals. There was a significant effect of treatment on body weight (F (2, 252) = 4.469, *p* < 0.05). However, there were no differences in the body weights of the animals in the different groups at any time.

### 3.2. Total Body Fat Mass

**Figure 2:**
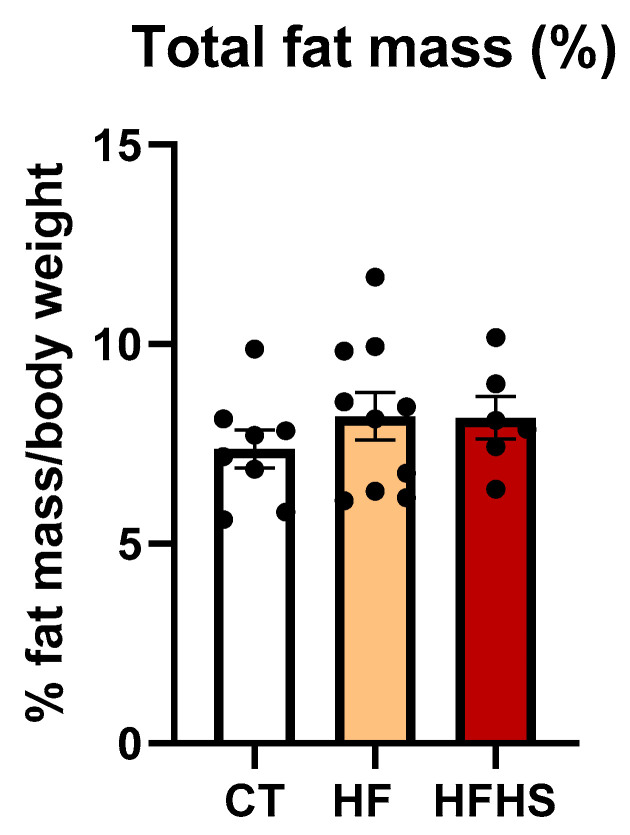
Histogram showing the mean ± SEM of the total fat mass percentage of control, HF, and HFHS animals. No significant differences were observed between groups. Dark circles represent the percentage of total fat mass per body weight of each animal.

The total body fat mass (Figure 2), expressed as a percentage of body weight (SEM), was 7.375 (0.483) for control rats, 8.191 (0.598) for HF rats, and 8.158 (0.535) for HFHS rats. As shown by ANOVA, there was no significant effect of dietary treatment on the total adipose tissue (F (2, 21) = 0.6714, n.s.). 

### 3.3. Chow Consumption

**Figure 3:**
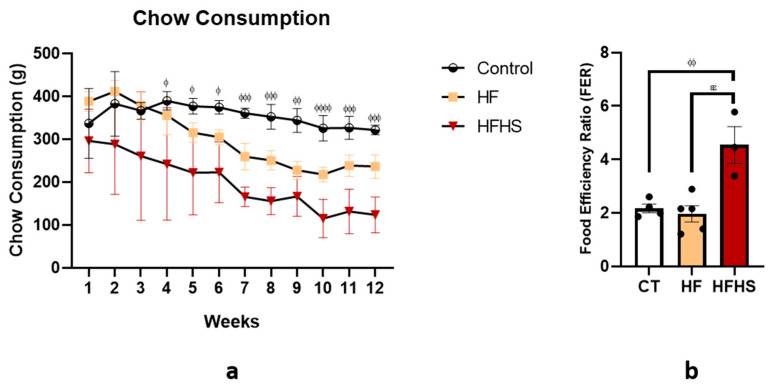
The graphic in (**a**) represents the food consumption during the twelve weeks of treatment. The histogram in (**b**) shows the Food Efficiency Ratio (FER) between the different groups. Data are presented as the mean ± SEM. ^ϕ^ *p* < 0.05; ^ϕϕ^ *p* < 0.01; ^ϕϕϕ^ *p* < 0.001; and ^ϕϕϕϕ^ *p* < 0.0001 HFHS versus controls; and ^εε^ *p* < 0.01 HFHS versus HF. Dark circles represent the Food Efficiency Ratio (FER) of each animal.

The animals chow consumption (Figure 3a) was recorded every week during all experiments to assess the regulation of appetite and eating behavior of the animals fed the three diets. Taking into account the Kcal of fat obtained from the diet, it ranges between 3% of fat in the normal chow and 20.1% of fat in the high-fat chow. The overall Kcal obtained directly from fat presented in diet were, on average, 420.9 Kcal for controls, 3283.3 Kcal for HF, and 4685.4 Kcal for HFHS animals. As shown by ANOVA, dietary treatment significantly influenced total chow consumption (F (2, 106) = 105.6, *p* < 0.001). The duration of the treatment also had a significant influence on chow consumption (F (11, 106) = 11.80, *p* < 0.001). HFHS animals consumed less chow than controls between weeks 4 and 12 (*p* < 0.05, weeks 4–6; *p* < 0.01, week 9; *p* < 0.001, weeks 7, 8, 10–12). We also assess the Food Efficiency Ratio (FER) (Figure 3b), and as demonstrated by ANOVA, the type of chow consumed (control or high fat) significantly influenced the FER (F (2, 9) = 12.60, *p* < 0.05). *Post-hoc* analysis showed that HFHS rats had a higher FER when compared to HF rats (*p* < 0.01) and control rats (*p* < 0.01). 

### 3.4. Liquid Consumption

**Figure 4:**
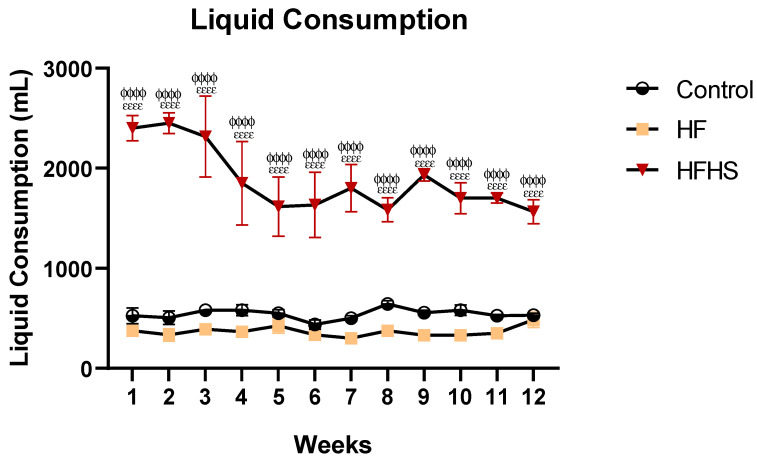
Graphical representation of liquid consumption during treatment. The control and HF groups consumed tap water, whereas the HFHS group consumed a 15% liquid sugar solution. Between weeks 1 and 12, HFHS animals consumed more liquid than the HF animals and controls. Data are presented as mean ± SEM. ^ϕϕϕϕ^ *p* < 0.0001 HFHS versus controls; ^εεεε^ *p* < 0.0001 HFHS versus HF.

Like chow, the quantity of liquid consumed, as shown in Figure 4, is an important measure because the HFHS group had a sucrose solution to drink, instead of tap water. As shown by the ANOVA, there was an effect of dietary treatment on liquid consumption (F (2, 107) = 633, *p* < 0.0001). HFHS rats consumed more liquid (liquid solution of 15% sucrose) than the HF (water, *p* < 0.0001) and control (water, *p* < 0.0001) rats. Although HF animals did not consume sucrose solution, the diet chow had sugar as a source of Kcal. The overall Kcal obtained from sugar was 2286.9 Kcal for HF and 6760.1 Kcal for HFHS animals. 

### 3.5. Behavioral Assessment

#### 3.5.1. Morris Water Maze (MWM)—Reference Memory

**Figure 5:**
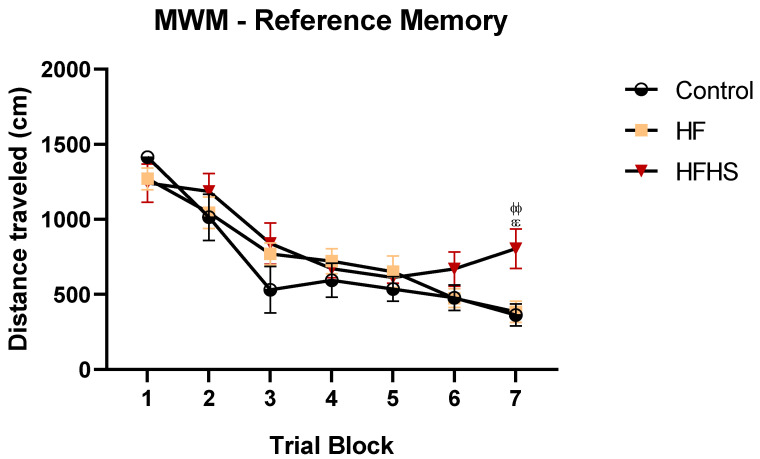
HFHS rats exhibit spatial learning and memory impairments. The graph shows the mean ± SEM total distance traveled to find the hidden platform for each block of trials in the Morris water maze. HFHS rats revealed impaired performance compared to HF and controlled animals in the last trial blocks. ^ϕϕ^ *p* < 0.01 HFHS versus controls; ^εε^ *p* < 0.01 HFHS versus HF group.

The MWM test is the most commonly used test for evaluating spatial memory and learning in rodents. To assess whether diet could alter these features, we performed three different MWM assessments: reference memory, working memory, and sensorimotor ability. Reference memory was used to evaluate long-term spatial memory and learning. The mean distances traveled to find a non-visible platform in the reference memory task of the MWM are shown in Figure 5. Repeated measures ANOVA showed that rats of all groups progressively reduced the distance traveled to locate the hidden platform during the acquisition days (F (6, 12) = 20.77, *p* < 0.0001), and a significant main effect of treatment on the distance traveled was also found (F (1.687, 10.12) = 6.484, *p* < 0.05). *Post-hoc* tests showed that the performance to locate the hidden platform during acquisition was significantly worse in HFHS rats than in HF rats (*p* < 0.01) and controls (*p* < 0.01) in trial block 7.

#### 3.5.2. Morris Water Maze (MWM) —Reference Memory: Probe Test

**Figure 6:**
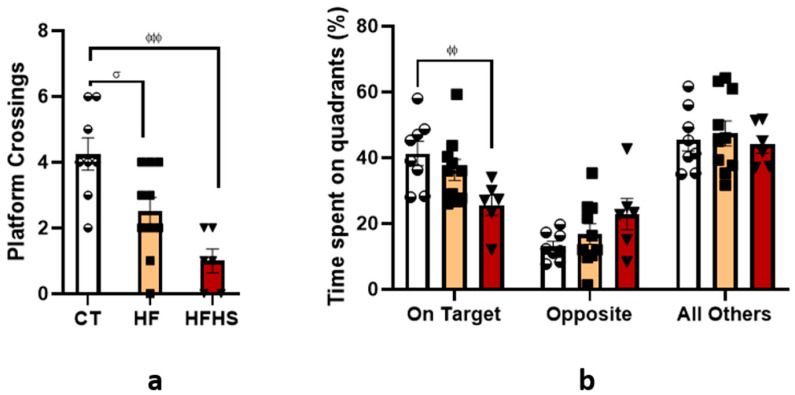
Histogram (**a**) shows the mean ± SEM number of platform crossings during the probe trial. HFHS rats crossed the former position of the platform less frequently than the control rats. The histogram in (**b**) shows the mean ± SEM values of the percentage of cumulative time spent throughout the test in the quadrant where the hidden platform was located compared to the opposite quadrant and all other quadrants. HFHS rats spent less time in the target quadrant than the control rats. ^ϕϕ^ *p* < 0.01, ^ϕϕϕ^ *p* < 0.001 HFHS versus controls; ^σ^ *p* < 0.05 HF versus control. The bars in white represent the mean for the control group, the bars in orange represent the mean for the HF group, and the bars in red represent the mean for the HFHS group. The circle represents the value for each animal in the control group, the square represents the value for each animal in the HF group, and the inverted triangle represents the value for each animal in the HFHS group.

The results of the analyses derived from the probe trial are shown in Figure 6. One-way ANOVA revealed a significant effect of treatment (F (2, 21) = 11, *p* < 0.001) on the number of platform crossings (Figure 6a). *Post-hoc* analysis showed that HFHS rats crossed the former position of the escape platform less frequently (*p* < 0.001) than control rats. The same was observed in HF rats (*p* < 0.05) compared to control animals. There were no differences between the HFHS and HF animals. Two-way ANOVA revealed a significant effect of the quadrant when it compared the target quadrant with the opposite quadrant and all other quadrants (F (2, 63) = 49.79, *p* < 0.0001) (Figure 6b). There was also a significant treatment × quadrant interaction (F (4, 63) = 3.116, *p* < 0.05). *Post-hoc* analysis showed that HFHS rats spent less time in the target quadrant than the control group (*p* < 0.01), but no differences between groups were found in the opposite quadrant and all other quadrants. 

#### 3.5.3. Morris Water Maze (MWM)—Working Memory

**Figure 7:**
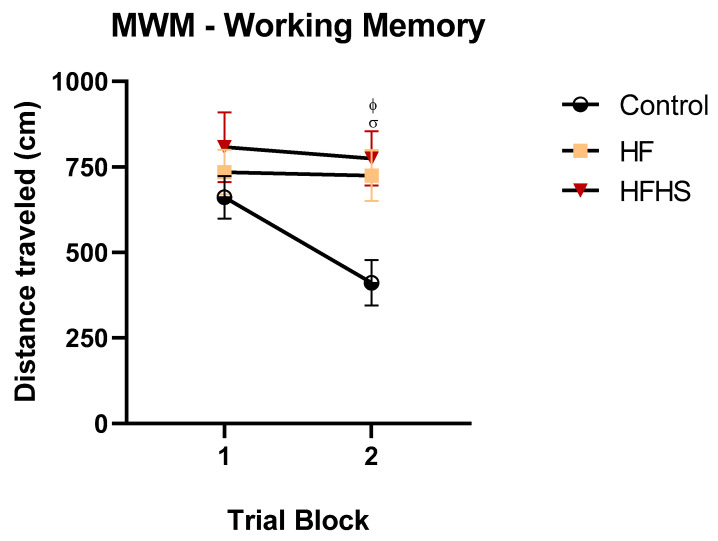
High-Fat High-Sugar, and High-Fat diets impair working memory. The graph illustrates the mean ± SEM of the distance traveled to find the hidden platform during the information and retention trials. HFHS and HF rats traveled long distances to find the platform in the retention trial than the controls. ^ϕ^ *p* < 0.05 HFHS versus control; ^σ^ *p* < 0.05, HF versus control.

Working memory variation in the MWM test was used to assess short-term spatial memory. Figure 7 shows the mean distances traveled to find the platform in the information and retention trials. Repeated measures ANOVA revealed that there was a significant effect of treatment (F (2, 20) = 6.05., *p* < 0.05.), in the trial-block effect (F (1, 20) = 5.20, *p* < 0.05) and the interaction (F (2, 20) = 3.37, *p* < 0.05. *Post-hoc* analysis showed that HFHS rats traveled long distances to find the platform in the retention trial compared to controls (*p* < 0.05) and the high-fat diet group (*p* < 0.05). 

#### 3.5.4. Open-Field

**Figure 8:**
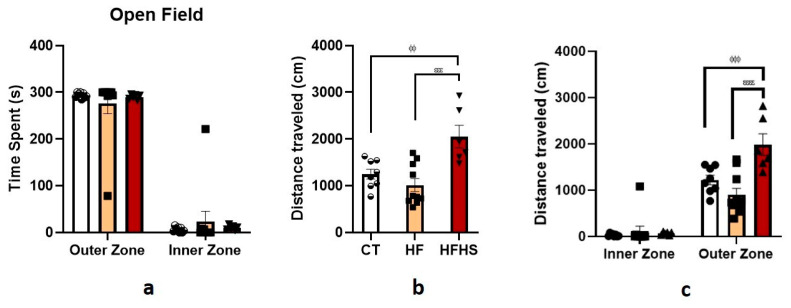
Open-field test. Histograms display the mean ± SEM time spent (**a**) in the outer and inner zones of the open field, the total distance traveled during all acquisitions (**b**), and the distance traveled in the outer and inner zones during the test (**c**). No differences were found among the three groups in the time spent in the inner and outer zones. For the total distance traveled, the HFHS animals traveled longer distances than the HF and control animals. For the distance traveled in the different zones of the apparatus, HFHS animals traveled long distances in the outer zone than HF and controlled animals; no differences were found for the inner zone. ^ϕϕ^ *p* < 0.01; ^ϕϕϕ^ *p* < 0.001 HFHS versus controls; ^εεε^ *p* < 0.001; ^εεεε^ *p* < 0.0001 HFHS versus HF. The bars in white represent the mean for the control group, the bars in orange represent the mean for the HF group, and the bars in red represent the mean for the HFHS group. The circle represents the value for each animal in the control group, the square represents the value for each animal in the HF group, and the inverted triangle represents the value for each animal in the HFHS group.

The open-field test assesses various aspects of rodents, such as locomotor activity, general activity levels, and exploration habits. For anxiety-like behavior, as shown in Figure 8a, the results showed that the time spent was significantly influenced by zone (F (1, 42) = 409.9, *p* < 0.0001) but not by dietary treatment (F (2, 42) < 0.0001, n.s.), with no treatment × zone interaction (F (2, 42) = 0.7172, n.s.). Rats of all groups spent more time (Figure 8a) in the outer zone than in the inner zone (*p* < 0.0001). Looking at the distance traveled (Figure 8b), it was observed that there was a significant effect of treatment (F (2, 42) = 8.667, *p* < 0.0001), zone (F (1, 42) = 163, *p* < 0.0001) and treatment × zone interaction (F (2, 42) = 10.01, *p* < 0.001). Locomotor activity was assessed as shown in Figure 8b,c. *Post-hoc* analysis revealed that HFHS-treated animals traveled more distance than control and HF rats in the outer zone (*p* < 0.001) but not in the inner zone, as presented in Figure 8c. The total distances traveled, expressed in centimeters, were 1249 cm for control rats, 1012 cm for HF rats, and 2050 cm for HFHS animals. As revealed by the ANOVA, diet significantly affected the total distance traveled in the open field (F (2, 21) = 10.61, *p* < 0.001). HFHS rats traveled longer distances than HF rats (*p* < 0.001) and control rats (*p* < 0.01). 

#### 3.5.5. Elevated Plus Maze

**Figure 9:**
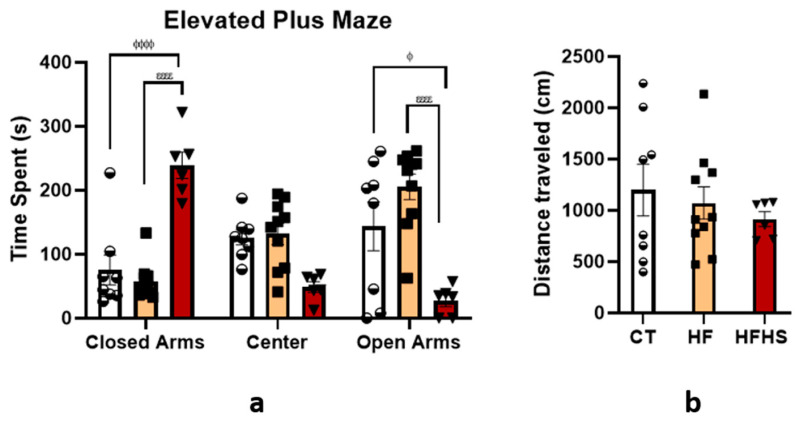
Elevated plus maze. The HFHS rats exhibited increased levels of anxiety. Histograms show the mean ± SEM time spent (**a**) in the arms and central square of the elevated plus maze and the total distance traveled during acquisition (**b**). HFHS rats spent more time in the closed arms of the maze compared to controls and HF rats. In agreement with this, they spent less time in the open arms of the maze compared to controls and HF rats. Furthermore, there were no significant differences in the distance traveled or time spent in the central square of the elevated plus maze between the three groups. No differences were found in the total distance traveled during acquisition. ^ϕ^ *p* < 0.05; ^ϕϕϕϕ^ *p* < 0.0001 HFHS versus control; ^εεεε^ *p* < 0.0001 HFHS versus HF rats. The bars in white represent the mean for the control group, the bars in orange represent the mean for the HF group, and the bars in red represent the mean for the HFHS group. The circle represents the value for each animal in the control group, the square represents the value for each animal in the HF group, and the inverted triangle represents the value for each animal in the HFHS group.

Like other types of mazes, the elevated plus test (Figure 9a) was used to assess anxiety-like behavior in rodents. The results show that neither zone (F (2, 63) = 1.190, n.s.) nor treatment (F (2, 63) = 1.326, n.s.) significantly influenced output. However, we found a significant interaction between treatment and zone (F (4, 63) = 21.21, *p* < 0.0001). *Post-hoc* tests revealed that HFHS rats spent more time in the closed arms than HF (*p* < 0.0001) and control (*p* < 0.0001) rats. Accordingly, the HFHS rats spent less time in the open arms than the HF (*p* < 0.0001) and control (*p* < 0.05) rats. In addition, it was observed that rats of the three groups expended more time in the closed arms than in the open arms of the apparatus. Locomotor activity was assessed, as shown in Figure 9b. The distances traveled, expressed in cm, were 1200 cm for control rats, 1072 cm for HF rats, and 915.4 for HFHS animals. As shown by the ANOVA, there was no significant effect of dietary treatment (F (2, 21) = 0.4865, n.s.). 

### 3.6. Neurogenesis—Doublecortin (DCX)

**Figure 10:**
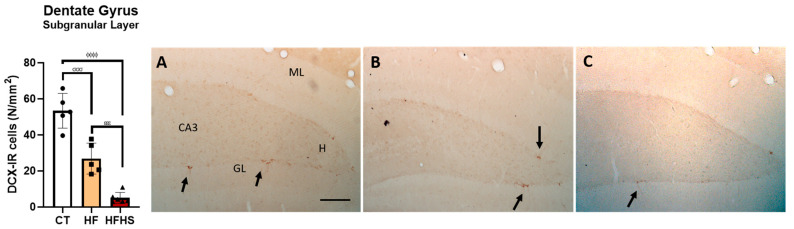
HFHS rats displayed decreased hippocampal neurogenesis. The histogram illustrates the mean ± SD of the total number of doublecortin (DCX)-IR neurons in the subgranular layer of the dentate gyrus. There was a significant reduction in the number of DCX-IR cells in HFHS animals compared to control and HF animals. ^ϕϕϕϕ^ *p* < 0.001 HFHS versus controls; ^σσσ^ *p* < 0.001, HF versus control; ^εεε^ *p* < 0.001 HFHS versus HF animals. The bar in white represent the mean for the control group, the bar in orange represent the mean for the HF group, and the bar in red represent the mean for the HFHS group. The circle represents the value for each animal in the control group, the square represents the value for each animal in the HF group, and the inverted triangle represents the value for each animal in the HFHS group. Illustrative photomicrographs of level-matched coronal sections of the dentate gyrus from control (**A**), HF (**B**), and HFHS (**C**) rats immunostained for doublecortin (DCX). Arrows show DCX-immunopositive cells in the dentate gyrus subgranular layer. ML, dentate gyrus molecular layer; GL, granule cell layer; H, dentate hilus; CA3, pyramidal cell layer of CA3 hippocampal field. Scale bar = 100 µm.

Estimations of the areal density of DCX-immunoreactive (DCX-IR) neurons in the subgranular layer of the HF are shown in Figure 10. The results showed a significant effect of treatment on the number of DCX-immunopositive neurons in the subgranular layer of the dentate gyrus (F (2, 13) = 48.90, *p* < 0.0001). *Post-hoc* analysis showed that HFHS animals had a significant reduction in the total number of DCX-IR neurons compared with HF (*p* < 0.001) and control animals (*p* < 0.001). HF rats also have fewer DCX-IR neurons than controls (*p* < 0.001).

### 3.7. Neuroinflammation—Astrocytes

**Figure 11:**
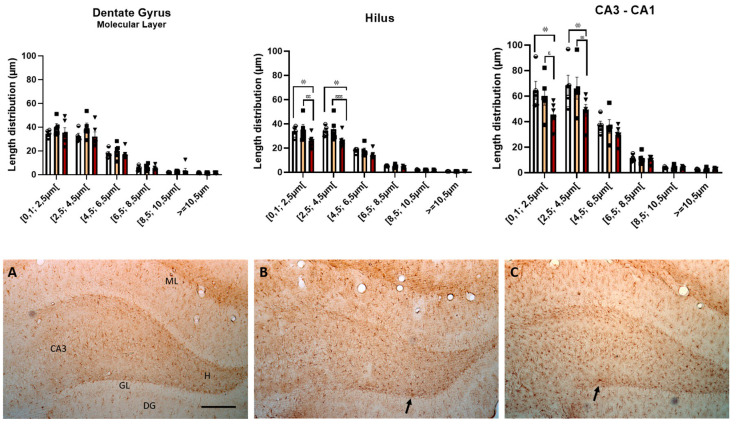
HFHS rats displayed decreased astrocyte processes. The histograms show the mean ± SD length distribution of astrocyte processes in the three different regions of the hippocampus. There was a significant reduction in processes with a minor length distribution compared to the control and HF animals. ^ϕϕ^ *p* < 0.01 HFHS versus controls; ^ε^ *p* < 0.05; ^εε^ *p* < 0.01; ^εεε^ *p* < 0.001 HFHS versus HF animals. The bars in white represent the mean for the control group, the bars in orange represent the mean for the HF group, and the bars in red represent the mean for the HFHS group. The circle represents the value for each animal in the control group, the square represents the value for each animal in the HF group, and the inverted triangle represents the value for each animal in the HFHS group. Representative photomicrographs of level-matched coronal sections of the hippocampus formation from control (**A**), HF (**B**), and HFHS (**C**) rats immunostained for Glial Fibrillary Acidic Protein (GFAP). Arrows show GFAP-immunopositive cells in the hippocampus formation. ML, dentate gyrus molecular layer; GL, granule cell layer; H, dentate hilus; DG, dentate gyrus; CA3, pyramidal cell layer of CA3 hippocampal field. Scale bar = 100 µm.

To assess whether the consumption of HF and HFHS diets by old animals (18 months old) can increase neuroinflammation, we investigated astrocytes in three different regions of the hippocampus. First, we analyzed the length distribution of astrocyte processes, as shown in Figure 11A–C. ANOVA revealed a significant effect of astrocytes projections length on the molecular layer of the dentate gyrus (F (5, 72) = 130.40, *p* < 0.0001), hilus (F (5, 72) = 162.2, *p* < 0.0001), and CA3-CA1 (F (5, 72) = 106.1, *p* < 0.0001), as well as a significant effect of dietary treatment for hilus (F (2, 72) = 8.112, *p* < 0.001) and CA3-CA1 (F (2, 72) = 5.886, *p* < 0.05), but no treatment × astrocytes projections length interaction in the molecular layer (F (10, 72) = 0.5107, n.s.), hilus (F (10, 72) = 1.644, n.s.), and CA3-CA1 (F (10, 72) = 1.357, n.s.). *Post-hoc* analysis revealed that the process length distribution was significantly smaller in the hilus region for HFHS animals, between 0.1 and 4.5, than in HF (*p* < 0.01) and controls (*p* < 0.05). The results also revealed that in the CA3-CA1 region, the HFHS animals also had a smaller distribution, between 0.1 and 4.5, than HF (*p* < 0.05) and controls (*p* < 0.01). These length distributions were unchanged for the higher length distributions in both regions. 

**Figure 12:**
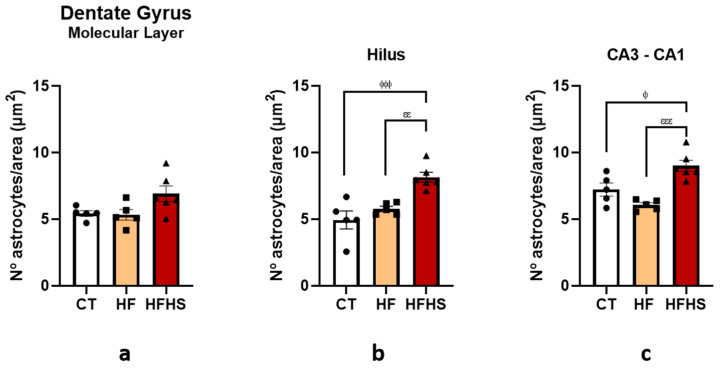
Histograms showing the mean ± SD number of astrocytes per area in the molecular layer (**a**), hilus (**b**), and CA3-CA1 (**c**) region. There was a significant increase in the number of astrocytes per area in the hilus and CA3-CA1 regions compared with the control and HF animals. ^ϕ^ *p* < 0.05; ^ϕϕϕ^ *p* < 0.001 HFHS versus controls; ^εε^ *p* < 0.01; ^εεε^ *p* < 0.001 HFHS versus HF animals. The bars in white represent the mean for the control group, the bars in orange represent the mean for the HF group, and the bars in red represent the mean for the HFHS group. The circle represents the value for each animal in the control group, the square represents the value for each animal in the HF group, and the inverted triangle represents the value for each animal in the HFHS group.

Beyond the length distributions of the astrocyte processes, we studied the number of astrocytes per area, as shown in Figure 12a–c, for the same regions. ANOVA revealed a significant effect of dietary treatment on the hilus (F (2, 12) = 12.46, *p* < 0.05) and CA3-CA1 (F (2, 12) = 13.84, *p* < 0.001) but not on the molecular layer (F (2, 12) = 3.470, n.s.). *Post-hoc* tests revealed that for the hilus, the number of astrocytes per area was higher in HFHS animals than in HF animals (*p* < 0.01) and controls (*p* < 0.001). The results also revealed that, for the CA3-CA1, the number of astrocytes per area was higher in the HFHS group than in the HF (*p* < 0.001) and control groups (*p* < 0.05).

**Figure 13:**
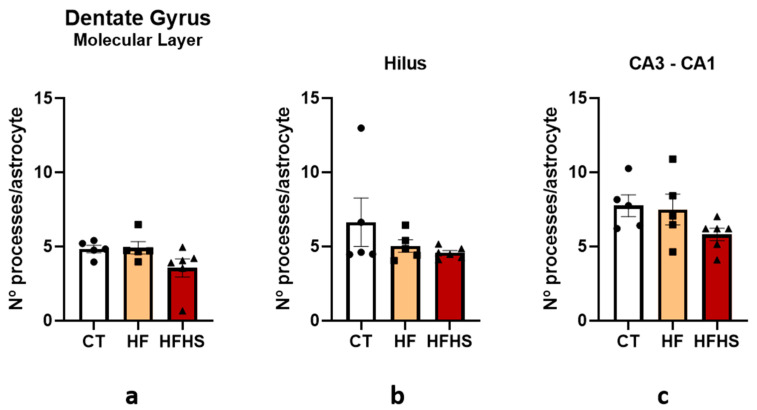
Histograms showing the mean ± SD number of processes per astrocyte in the molecular layer (**a**), hilus (**b**), and CA3-CA1 (**c**) region of the hippocampus. There were no significant differences among the three groups. The bars in white represent the mean for the control group, the bars in orange represent the mean for the HF group, and the bars in red represent the mean for the HFHS group. The circle represents the value for each animal in the control group, the square represents the value for each animal in the HF group, and the inverted triangle represents the value for each animal in the HFHS group.

Because there were statistical differences in the number of astrocytes per area in two of the three regions studied, we decided to analyze the morphology of astrocytes, as shown in Figure 13a–c, by counting the number of processes per astrocyte. A two-way ANOVA revealed no significant effect of dietary treatment on the molecular layer (F (2, 12) = 2.368, n.s.), hilus (F (2, 12) = 1.103, n.s.), or CA3-CA1 (F (2, 12) = 1.882, n.s.). We found no difference in the number of processes per astrocyte between groups for all three regions. However, we observed a slight decrease in the number of processes in HFHS astrocytes in the three areas. 

## 4. Discussion

The main goal of the current study was to compare the effects of two different high-caloric diets, namely, high-ft, and high-fat-high-sugar diets, on aged Wistar rats. Our findings demonstrated that, in aged rats, the HFHS diet was generally more deleterious than the HF diet. Indeed, we found that the HFHS diet impaired spatial learning and memory and working memory and increased anxiety-like behavior, which was associated with decreased neurogenesis and increased neuroinflammation in the hippocampus. Interestingly, in the present study, we also observed that the high-fat diet impaired spatial and working memory, but the severity of the alterations was considerably lower than that observed in high-fat, high-sugar diets. 

Body weight analysis revealed that the consumption of high-caloric diets did not result in a significant increase in the mean body weight. This result was not surprising because former studies demonstrated that high-caloric diets might not cause a significant increase in body weight during the juvenile [33,34,35] and adult periods, such as middle age [6,14]. However, careful inspection of the body weight graph clearly showed a tendency towards an increase in body weight, which was proportionally higher in HFHS rats, suggesting that if we continued the administration of the HFHS diet, we would have significant differences in weight. Interestingly, no increase in body fat mass was observed in either the HF or HFHS group, even after 12 weeks of treatment. We have previously found that in young animals, high-caloric diets could induce functional impairments, even without a significant increase in body mass [35]. Corroborating this view that a high-caloric diet induces brain impairments before obesity establishment [36], in the present study, we found that, in aged rats, high-caloric diets induced functional alterations, even without significant changes in body weight and body fat mass. 

In addition to the absence of changes in body weight and mass during the current high-caloric diet, it is important to analyze the energy source. Thus, the energy consumed by HF rats was like that observed in the control rats because, although high-fat chow had more calories than standard chow, high-fat rats consumed less. Conversely, in the HFHS group, we observed an increase in the number of calories ingested compared to both control and HF rats. Looking more deeply at this, we can see that this increase in calories in HFHS rats was mainly due to the consumption of the high-sugar solution since the consumption of chow was lower than that observed in the controls. HFHS animals consumed, on average, an overall of 23,311 kcal, of which 4685.4 kcal were obtained from fat and 6760.1 kcal from sugar. On the other hand, HF animals consumed 16,335 kcal, of which 3283.3 kcal were obtained from fat, and 2286.9 kcal were obtained from sugar. Control animals consumed 14,032 kcal, of which 420.9 kcal were obtained from a fat source. This information is crucial for interpreting the behavioral and morphological results, which we will now discuss. 

Our results showed that the HF diet did not affect locomotor activity, revealing that this diet did not significantly impair any of the neural structures implicated in the locomotor behavior. However, animals that ingested the HFHS diet were observed to travel more distances than the controls in the open-field, which was only significant in the outer zone. Because there was no significant increase in the distance traveled in the unprotected inner zone, which is particularly sensitive to anxiolytic effects, we think that these alterations in HFHS rats are more related to an increase in exploratory activity than to a decrease in anxiety levels [37]. 

Relative to anxiety, we observed that HF and HFHS treatments did not affect the time animals spent in the inner and outer zones of the open-field. However, anxiety-like behavior was observed in HFHS-fed rats, as they spent more time in the closed arms of the elevated plus-maze compared to control and HF animals. Confirming this anxiety-like behavior, it was also observed that HFHS significantly reduced the time spent in the open arm of the apparatus. These results corroborate a previous study showing the anxiogenic effect of a high-caloric diet in older male rats [38]. However, it is necessary to take into account that these results, and the behavioral anxiety tests used, reflect a small fraction of the emotional state that was assessed and did not reflect the entire emotional prism of the animals. In addition, our results also seem to show that the elevated plus-maze test was, in this case, more sensitive to anxiety-like behavior than the open-field test. It is important to note that this emotional behavior is related to the diet consumed, as the animals maintained their group-specific diet for 12 weeks, including behavioral assessments. Therefore, the anxiogenic effect could not be related to the possible withdrawal of access to food or beverages. It is also important to note that no anxiety-like behaviors were observed in our HF animals since the results were like those observed in the control animals. Thus, it seems that the moderate increase in fat caloric consumption was not enough to induce anxiety-emotional alterations, even in a susceptible period as it is in the elderly. Conversely, the consumption of higher amounts of calories from fat and refined sugar induces changes in anxiety in aged animals.

Relative to cognition, our results showed that the HFHS diet impaired spatial learning and memory in aged rats, corroborating previous studies that used high-caloric diets [2,3,6]. Indeed, HFHS rats had a worse performance during the acquisition phase of the spatial version of the Morris water maze, and this difference was significant at the last trial block compared to control rats. Furthermore, the impairment observed throughout the acquisition phase had consequences in the spatial memory evaluated in the probe trial on day 15, as shown by the lower platform crossings of the HFHS animals and by the fact that HFHS animals spent less time in the target quadrant than HF and control animals. Reinforcing the information regarding the deleterious impact of HFHS on cognition, we also found a significant impairment of the working memory process in HFHS-aged animals, corroborating previous studies using high-caloric diets in middle-aged rats [12,17,39]. Interestingly, relative to the HF diet, we found that our HF diet did not significantly impair spatial learning processes but was indeed able to impair spatial memory and working memory. Together, these findings corroborate the hypothesis that high-caloric diets may impair cognitive processes before the onset of obesity, even in the elderly, supporting the observations of previous studies that HFHS rats exhibit cognitive impairment and anxiety-like behaviors even in the absence of obesity [16,35]. Furthermore, these results also align with previous findings that hippocampal plasticity could suffer direct deficits from carbohydrates and fats, independent of peripheral metabolic alterations such as weight [40]. Indeed, these findings reinforce the opinion that the elderly is a period of high vulnerability to the neural substrate that mediates hippocampal-dependent cognitive processes in certain high-caloric diets [6,12,14]. Moreover, the differential effects of HFHS and HF suggest that the possible detrimental effects of high-caloric diets on cognition are multifactorial and depend on diet composition, treatment duration, and age at which these diets are continuously consumed. Knowing that the hippocampus is crucial for reference and working memory [35,41], our results indicate that the consumption of high-caloric diets during aging can significantly affect the hippocampus. 

These deleterious effects of high-caloric diets on hippocampal-dependent functions can be explained by several mechanisms, including changes in neurogenic processes and neuroinflammation [33,42,43]. Furthermore, the elderly is critical for the survival of neural stem cells in the dentate gyrus of the hippocampus, and it is known that aging leads to a severe decline in the number of these precursors of new neurons and, consequently, spatial learning and memory as well as anxiety-like behavior [44]. Corroborating this view and considering that the present HFHS diet impairments learning and memory, our results showed that the density of DCX-positive cells in the hippocampus was significantly lower in HFHS rats than in both HF and control groups, indicating that the HFHS diet decreases neurogenic processes in aged rats. Therefore, it is conceivable that the enhanced anxiety levels and impairment of spatial learning and memory in HFHS-treated rats might be attributed to decreased neurogenesis in the hippocampus. This is supported by previous studies showing that impairment of neurogenesis induces an increase in anxiety behaviors [45,46] and cognitive dysfunction [47,48]. However, it is also important to note that other studies have shown that the ablation of new neurons from the adult hippocampus is associated with improved working memory performance [49] and preserved spatial reference memory [50]. Thus, it seems that the newly produced neurons could participate in part, but not all, of the hippocampal functions. Their specific role may vary, depending on several factors, such as the nature and cognitive demands of the tasks [49]. Curiously, albeit to a lesser extent than HFHS, HF induced a significant reduction in the number of DCX-IR cells in the hippocampus. Comparing the results, it is interesting to note that there is an association between the degree of severity of behavioral impairment and the degree of reduction of neurogenesis. Indeed, HFHS rats had considerable alterations in anxiety, spatial learning, and memory, associated with a considerable reduction in neurogenesis. In contrast, in HF rats, a lesser extent of behavioral impairment was also associated with a lesser reduction of the neurogenic process.

Naturally, other mechanisms are involved in the deleterious effects of high-caloric diets, including neuroinflammation [51]. Astrocytes are glial cells that interact intimately with neurons and ensure they receive, process, and propagate information to other neurons. In addition, astrocytes promote neurogenesis [52,53]. However, astrocytes become activated after brain injury and shift their function, contributing to age-related inflammatory processes, decreased neurogenesis, and neurodegeneration [54]. Contributing to this view, our results showed that the HFHS diet increased neuroinflammation through astrocytosis, suggesting diet-induced inflammation in the dentate gyrus and CA3-CA1 areas of the hippocampus, as it has already been described in other studies [8,15,55]. Indeed, we found that HFHS-treated rats had more GFAP-positive astrocytes per area; however, these astrocytes had fewer projections than those of the control and HF animals, which is not in line with classical astrocytosis morphology, where the projections become longer and less abundant [56]. The increase in the intermediate filament marker GFAP was in line with that described for astrocytosis in HFHS animals [57,58]. Indeed, previous studies have shown that ablation of GFAP markers from astrocytes leads to an increased and permissive environment for neuronal stem/progenitor cell differentiation since astrocytes without GFAP became less differentiated be more able to support regeneration and neurogenesis [58,59]. It has been reported that in old mice, this ablation leads to a 30% improvement in neurogenesis in the dentate gyrus of the hippocampus [59], one of the areas with increased neuroinflammation and decreased neurogenesis in our HFHS animals. Thus, it is tempting to suggest that the increase in GFAP-positive astrocytes in the hippocampus of HFHS animals may be related to the reduction in neurogenesis and, consequently, to the impairment of cognition and emotions [60].

On the other hand, the altered morphology with shortening and simplification of astroglial processes in the hippocampus may also explain, by itself, the impaired cognition and memory of HFHS animals, as previous studies have shown that the generation of specific astrocyte populations in the hippocampus, leads to severe disturbances in spatial reference memory [61,62]. In our study, if they just became activated, they could probably be A1 astrocytes, since this, in aging, enhances susceptibility to neurodegeneration and causes the loss of many functions that promote neuronal survival [62]. However, we can also consider that although under astrocytosis, they could start to defeat and shrink. To verify this, we believe that astrocytes may experience a mechanism of cell death, such as apoptosis, pyroptosis, or even senescence, as reported in other studies [63,64]. At the same time, gliogenesis may also occur because stem cells that become neurons can also become astrocytes, especially during aging [20]. Thus, gliogenesis increase the number of astrocytes to act as a repair mechanism. However, further studies are needed to answer these questions accurately. 

It is known that high-caloric diets induce deleterious alterations in the brains of juveniles and adults; however, information about the effects of these kinds of diets on the elderly is scarce. In this way, the present work contributes to the state of the art of the impact of diets on the brain by demonstrating that high-caloric diets induce several deleterious alterations in the hippocampus of old animals, showing the high susceptibility of aged rats to high-caloric diets. Another subject addressed in our study was the duration of treatment. Many studies on diet have been based on short treatments. To complement this knowledge, our study used a chronic treatment model that better mimicked the consumption of high-caloric diets in modern societies. Furthermore, it is important to note that the present high-caloric diets started to be administered at the beginning of old age; thus, the consumption of caloric diets in the elderly, even after an entire life with healthy habits, is sufficient to induce impairments in emotion and cognition.

Moreover, even a moderately high-fat diet was sufficient to induce cognitive alterations in aged rats, showing the brain susceptibility of aged individuals to high-caloric diets. Another contribution of this study is the comparison between two different high-caloric diets that are commonly consumed in modern societies. It was interesting to observe the different effects of HFHS and HF diets on the brains of aged rats. Although both are considered high-caloric diets, the rats of the present study consumed more calories in the HFHS diet than in the HF diet. Thus, the higher number of calories ingested by the HFHS rats resulted in a higher magnitude of behavioral changes in cognition and emotion than the changes induced by the moderate high-caloric HF diet. Similarly, the high number of calories in the HFHS diet induced more deleterious alterations in neurogenesis and neuroinflammation than in the HF diet. 

## 5. Conclusions

Our results show that in aged rats, the HFHS diet impairs spatial learning, memory, and working memory and increases anxiety-like behavior, which is associated with a decrease in neurogenesis and an increase in neuroinflammation in the hippocampus. The HF diet impaired spatial and working memory and reduced neurogenesis; however, the severity of the alterations was considerably lower than that observed in high-fat, high-sugar diets, demonstrating that, in aged rats, the HFHS diet is generally more deleterious than the HF diet. Thus, our results show that aged rats are highly susceptible to high-caloric diets, even if the diets are only started in the elderly, with an impact on cognition and emotions.

## Figures and Tables

**Table 1:** Composition of experimental diets.

		Control	High-Fat	High-Fat High-Sugar
Diet		Mucedola 4RF1	ssniff® E15749	ssniff® E15749 + 15% sucrose solution
Chow %/100g	Proteins	18.5	21.2	21.2
	Fibers	6	5.5	5.5
	Fat	3	20.1	20.1
	Carbohydrates			
	Starch	42.63	19.2	19.2
	Sucrose	3.68	14	14
Liquid Solution %/100g	Sucrose	0	0	15
Total Energy (Kcal/100g)		387.6	435.5	493.4

**Table 2:** Characteristics of rats fed with control, high-fat, high-fat, high-sugar diets for 12 weeks.

	Control	High-Fat	High-Fat High-Sugar
n	8	10	6
Initial Body Weight (g)	698.6 ± 17.23	641.3 ± 14.11	664.8 ± 32.17
Body Weight Gain (g)	61.63 ± 8.87	75.80 ± 8.33	110.3 ± 38.61
Total Fat mass (%)	7.37 ± 0.48	8.191 ± 0.59	8.158 ± 0.53
Chow Consumption (average/week) (g)	354.8 ± 6.86	298.7 ± 20.14	199.1 ±18.58
Liquid Consumption (average/week) (mL)	543.2 ± 15.05	366.3 ± 14.44	1879 ± 94.44
Total Energy (Kcal/100 g)	14032 ± 546.20	16335 ± 411.7	23311 ± 3453

## Data Availability

Not applicable.
